# A Regression Model to Predict Augmented Renal Clearance in Critically Ill Obstetric Patients and Effects on Vancomycin Treatment

**DOI:** 10.3389/fphar.2021.622948

**Published:** 2021-06-11

**Authors:** Lian Tang, Xin-yuan Ding, Lu-fen Duan, Lan Li, Hao-di Lu, Feng Zhou, Lu Shi, Jian Lu, Yi Shen, Zhi-wei Zhuang, Jian-tong Sun, Qin Zhou, Chen-qi Zhu, Jing-jing Li, Yan-xia Yu

**Affiliations:** ^1^ Department of Pharmacy, The Affiliated Suzhou Hospital of Nanjing Medical University, Suzhou Municipal Hospital, Suzhou, China; ^2^ Department of Obstetrics and Gynecology, The Affiliated Suzhou Hospital of Nanjing Medical University, Suzhou Municipal Hospital, Suzhou, China; ^3^ Intensive Care Unit, The Affiliated Suzhou Hospital of Nanjing Medical University, Suzhou Municipal Hospital, Suzhou, China; ^4^ Good Clinical Practice Office, The Affiliated Suzhou Hospital of Nanjing Medical University, Suzhou Municipal Hospital, Suzhou, China

**Keywords:** augmented renal clearance, critically ill obstetric patients, regression model, vancomycin, population pharmacokinetic model

## Abstract

**Background:** Augmented renal clearance (ARC) risk factors and effects on vancomycin (VCM) of obstetric patients were possibly different from other populations based on pathophysiological characteristics. Our study was to establish a regression model for prediction of ARC and analyze the effects of ARC on VCM treatment in critically ill obstetric patients.

**Methods:** We retrospectively included 427 patients, grouped into ARC and non-ARC patients. Logistic regression analysis was used to analyze the factors related to ARC. Receiver operating characteristic (ROC) curve was drawn to evaluate the predictive value of the model for ARC. Patients who received VCM therapy were collected. The published VCM population pharmacokinetic (PPK) model was used to calculate pharmacokinetic parameters. A linear regression analysis was made between the predicted and measured concentrations.

**Results:** Of the 427 patients, ARC was present in 201 patients (47.1%). The independent risk factors of ARC were heavier, greater gestational age, higher albumin level, fewer caesarean section, severe preeclampsia and vasoactive drug; more infection, hypertriglyceridemia and acute pancreatitis. We established the above nine-variable prediction regression model and calculated the predicted probability. ROC curve showed that the predicted probability of combined weight, albumin and gestational age had better sensitivity (70.0%) and specificity (89.8%) as well as the maximal area under the curve (AUC, AUC = 0.863). 41 cases received VCM; 21 cases (51.2%) had ARC. The initial trough concentration in ARC patients was lower than in non-ARC patients (7.9 ± 3.2 mg/L vs 9.5 ± 3.3 mg/L; *p* = 0.033). Comparing the predicted trough concentration of two published VCM PPK models with the measured trough concentration, correlation coefficients (*r*) were all more than 0.8 in the ARC group and non-ARC group. AUC was significantly decreased in the ARC group (*p* = 0.003; *p* = 0.013), and clearance (CL) increased in the ARC group (*p* < 0.001; *p* = 0.008) when compared with the non-ARC group.

**Conclusion:** ARC is a common state in critically ill obstetric patients. The regression model of nine variables had high predictive value for predicting ARC. The published VCM PPK models had good predictive performance for predicting trough concentrations of obstetric patients. Pharmacokinetic parameters of VCM are different in ARC obstetric patients, which results in enhanced VCM clearance and decreased trough concentration.

## Introduction

Augmented renal clearance has been defined as creatinine clearance [(CrCl) > 130 ml/min/1.73 m^2^] (Bilbao-Meseguer et al., 2018). It refers to the enhanced elimination of solutes by the kidneys, and is common in critically ill patients. The risk factors for ARC included younger age, diagnosis of polytrauma, and lower illness severity (Bilbao-Meseguer et al., 2018). ARC is a risk factor for subtherapeutic concentrations of β-lactam antibiotics and vancomycin regardless of using the standard dosage (Huttner et al., 2015; Hirai et al., 2016; Bilbao-Meseguer et al., 2018). However, the risk factors and prediction model of ARC vary in different populations. ARC risk factors of obstetric patients are possibly different from those of other populations based on pathophysiological characteristics. The incidence, predictive factors, and effect on antibiotics of ARC in critically ill obstetric patients are still unclear.

Critically ill obstetric patients include patients with severe pancreatitis, acute fatty liver, eclampsia, HELLP syndrome, shock, disseminated intravascular coagulation (DIC), postpartum hemorrhage, amniotic fluid embolism, intrahepatic cholestasis syndrome, and so on during pregnancy and obstetric care (Say et al., 2009). Pregnant patients have undergone a series of physiological changes with the increase of gestational age and actual weight gains: the protein binding rate decreases, the apparent volume of distribution becomes larger, and the liver metabolism increases (Anderson, 2005). Theoretically, the critically ill obstetric patients are younger and heavier, and the incidence of ARC may be higher than that in the general population. Risk factors of ARC in obstetric patients are different from other populations, so it is necessary to establish an ARC prediction model for identifying ARC in obstetric patients.

VCM is the main therapy for severe infections of methicillin-resistant *staphylococcus aureus* (MRSA) and *coagulase negative staphylococcus* (Rybak et al., 2020). Currently, VCM is widely used in critically ill obstetric patients; no cases of congenital defects have been observed during VCM therapy (Bookstaver et al., 2015; Briggs et al., 2017; Rac et al., 2018). According to the guidelines of therapeutic drug monitoring (TDM) of VCM (Rybak et al., 2020), the VCM trough concentration is recommended to be 10–20 mg/L, and AUC_24 h_/MIC at 400–600 as the best predictor of successful treatment of MRSA infections and low nephrotoxicity when the MIC ≤1 mg/L. Some studies have shown that the ARC status affected the serum concentration of VCM; although the dose of VCM was higher (>2 g/day), the concentration of VCM was lower (<15 mg/L) in ARC patients (Chu et al., 2016; Hirai et al., 2016; Tang et al., 2018a; Tang et al., 2018b). The physiological changes of critically ill obstetric patients lead to a high clearance of drugs, such as VCM, eliminated by the kidneys. These changes may theoretically lead to a decrease in the serum concentration of VCM, resulting in poor clinical efficacy or induction of bacterial resistance. It is necessary to analyze how to optimize the dosage regimen of vancomycin in obstetric patients. The purposes of our study were to establish a regression model for prediction of ARC in critically ill obstetric patients, and to evaluate population pharmacokinetics (PPK) software for predicting VCM concentration, and to provide reference for VCM optimizing administration in ARC obstetric patients.

## Methods

### Study Design, Population, and Data Collection

This retrospective observational study was performed in critically ill obstetric patients hospitalized in the Maternal Intensive Care Unit (MICU) from July 2016 to December 2018 at the Affiliated Suzhou Hospital of Nanjing Medical University. This study was approved by the research ethics committee of this hospital (K2016024). Patient data were obtained from the Perinatal Medical Database in July 2020. Clinical diagnoses were based on the 10th clinical modification revision of the International Classification of Diseases (ICD-10-CM). Pregnant women ≥20 weeks of gestational age enter the perinatal period, according to the American Association of Obstetricians and Gynecologists (American College of Obstetricians and Gynecologists and Committee on Practice Bulletins-Obstetrics, 2012). Inclusion criteria: The criteria for critical ill obstetric patients were in accordance with international definition of critical ill obstetric patients (Say et al., 2009). The enrolled critical ill obstetric patients were needed advanced life supports for any of the following reasons: respiratory failure, pulmonary embolism, severe preeclampsia with poor control of hypertension, eclampsia, HELLP syndrome, severe postpartum hemorrhage with unstable vital signs (bleeding volume >1,500 ml or a decrease in the hemoglobin concentration >40 g/L or acute hemorrhage >4 U), cerebral hemorrhage or cerebral infarction, DIC, severe infections, shock, arrhythmia requiring cardioversion or defibrillation, severe acute pancreatitis and acute fatty liver requiring vasoactive drugs to maintain stable hemodynamics, intubation and mechanical ventilation, or continuous renal replacement therapy or plasmapheresis in the MICU. Exclusion criteria were: (1) Patients with incomplete or missing clinical and laboratory information, such as having no serum creatinine level, Acute Physiological and Chronic Health Evaluation II (APACHE II) score, and so on during MICU hospitalization; (2) ICU stay less than 24 h.

On admission, demographic data (age, weight, height, gestational age), and primary diagnostic categories and basic diseases (eclampsia, HELLP syndrome, severe obstetric hemorrhage, and other severe obstetric complications) were registered. APACHE II, albumin, alanine aminotransferase, creatinine and other laboratory indicators, cesarean section, infection, trauma, mechanical ventilation, or the use of vasopressors were recorded.

### Calculation of Estimated Glomerular Filtration Rate (eGFR) and Groups

ARC was defined as CrCl ≥ 130 ml/min/1.73 m^2^, preferably measured in urine (Bilbao- Meseguer et al., 2018), but urine creatinine was not determined in these retrospectively collected obstetric patients. We used glomerular filtration rate estimating equations in the diagnosis of critically ill patients with ARC (Bilbao-Meseguer et al., 2018). Compared with other eGFR calculation formulas (CG formula, MDRD formula), many research studies have indicated that the Chronic Kidney Disease Epidemiology Collaboration (CKD-EPI) equation had better performance to detect ARC (Baptista et al., 2014; Ruiz et al., 2015; Wu et al., 2019; Gijsen et al., 2020). In our study, 24 h urinary CrCl of patients had not been measured, so we calculated eGFR according to the CKD-EPI equation. Patients were divided into the following groups: ARC group with eGFR >130 ml/min/1.73 m^2^, and non-ARC group with eGFR ≤130 ml/min/1.73 m^2^. The creatinine concentrations in plasma were measured by the creatininase-HMMPS method (L-Type creatinine M; Wako Pure Chemical Industries, Ltd.) with the automated analyzer. Since all the patients we included were female, we used the CKD-EPI equation of female taking into account Scr, gender and age as follows:

Serum creatinine µmol/l (mg/dl) ≤ 62(≤0.7):

$$eGFR=144\times {\left(\frac{Scr}{0.7}\right)}^{-0.329}\times 0.993^{age}.$$ (1)

Serum creatinine µmol/l(mg/dl) > 62(>0.7):

$$eGFR=144\times {\left(\frac{Scr}{0.7}\right)}^{-1.209}\times 0.993^{\text{age}},$$ (2)

Scr(µmol/l)=88.4×Scr(mg/dl), (3)

where *Scr* is the serum creatinine from the first or second day of admission.

### Establishment of Regression Model

Univariate and multivariate logistic regression analysis was used to select the covariables of the regression model. In the univariate analyses, the χ2 test or Fisher exact test was used to compare the categorical variables. The continuous variables were compared with the Mann-Whitney *U* test. The covariates with *p* values of <0.1 were included in the multivariate logistic regression analysis (backward procedure, based on *p*-value of predictor removed) (Hammitt et al., 2019). Multivariate logistic regression analysis was used to screen for independent risk factors. SPSS software automatically screened out the independent variables when the optimal balance was reached between the fitting degree of the prediction model.

Independent risk factor variables were used to establish regression equations and calculate prediction probabilities. The receiver operating characteristic (ROC) curves of multivariate factors were drawn; the area under the curve (AUC), cut-off point, Youden index, sensitivity, and specificity were used to evaluate their predictive value for predicting ARC.

### Clinical Validation of the Regression Model

We retrospectively selected critically ill obstetric patients who received VCM for suspected or confirmed gram-positive infections and measured the steady-state trough concentration (after the fourth maintenance dose, and 30 min prior to the next dose) from January 2019 to June 2020. According to the eGFR (CKD-EPI equation), patients were grouped into ARC patients and non-ARC patients. The prediction probability for each patient was calculated based on the regression model, and the cut-off point of prediction probability was used to determine whether the patients had ARC status. The predictive accuracy of this logistic regression model for ARC prediction was expressed as positive prediction accuracy and negative prediction accuracy.

### The Effect of ARC on VCM Trough Concentration

The target serum VCM trough concentrations range from 10 to 20 mg/L, AUC_24 h_ at 400–600 (MIC = 1). The serum trough concentrations of VCM were measured by chemiluminescent enzyme immunoassay. The automatic chemiluminescence immunoassay (ARCHITECT I2000SR), VCM kit, and quality control reagent were all products of Abbott Company, Illinois, United States. The dosage, period, and trough concentration of vancomycin were compared between the ARC group and non-ARC group.

### Calculation of VCM Pharmacokinetic Parameters

We calculated the apparent volume of distribution (Vd), clearance rate (CL), and AUC_24 h_ of VCM in critically ill obstetric patients using PPK software (Bayesian method) *JavaPK for Desktop* (*JPKD*)-vancomycin *Ver. 3.1* and *SmartDose. JPKD* is a clinical pharmacokinetic (CPK) services computer program for desktop (Download: http://pkpd.kmu.edu.tw/jpkd/). *JPKD* was developed by Lee et al. From the College of Pharmacy, Kaohsiung Medical University in Taiwan. The published PPK model of *JPKD-vancomycin* in this software was based on the analysis of vancomycin in patients with hematological malignancies (Buelga et al., 2005). *SmartDose* is a decision support system for individualization of vancomycin dosage. It was developed using the maximum a posterior Bayesian estimation (MAPB) by the open-source language R combined with the population PK characteristics of vancomycin in Chinese patients. *SmartDose* has high computational reliability, which is validated by NONMEM, the gold standard PK software. Meanwhile, *SmartDose* is established as a web-based application, and its operational flexibility makes it an efficient tool for vancomycin dose optimization in routine clinical settings (Gao et al., 2018). Many studies have indicated that *JPKD-vancomycin* and *SmartDose* had good prediction for the samples from Chinese population (He and Yang, 2015; He et al., 2016; Fang et al., 2017; Tang et al., 2018b; Lin et al., 2019; Ni et al., 2019; Xue et al., 2020). The pharmacokinetic parameters of VCM calculated by *JPKD* and *SmartDose* were similar to literature reports (Hirai et al., 2016). The Vd and Cl of the patient can be calculated by inputting the patient's gender, weight, and creatinine into the software. The AUC_24 h_ calculation formula is as follows:

$${\text{AUC}}_{24\text{h}}=\frac{\text{daily}\;\text{dose}\left(mg\right)}{CL\left(L/h\right)}.$$ (4)

### Predictive Performance of Vancomycin PPK Software

The precision of the PPK software was assessed by calculating the median absolute prediction error (APE) for the initial trough concentration according to Eq. 5. Linear regression models were used to compare predicted and measured vancomycin initial trough concentrations.

$$\text{Absolute}\;\text{prediction}\;\text{error}\left(\text{precision}\right)=\frac{\left|\text{Cp}-\text{Cm}\right|}{\text{Cm}}\times 100\%,$$ (5)

where Cp refers to the model predicted vancomycin concentration and Cm refers to the measured vancomycin concentration.

### Statistical Analysis

All statistical analyses were performed using the Statistical Package for Social Sciences, version 22 (SPSS Inc. Chicago, IL, United States), and GraphPad Prism version 6. The categorical variables are summarized as frequencies and proportions (%); Pearson’s chi-square test or Fisher’s exact test was used to analyze categorical data. All the continuous variables were checked for normality using the Shapiro-Wilk test. The continuous variables are summarized as the medians and interquartile ranges; Mann-Whitney *U* test was used to analyze continuous data when these data were not normally distributed. Continuous variables which are in accordance with normal distribution are summarized as χ ± SD; *t*-test was used to analyze these continuous data. Two-tailed *p* values of <0.05 were considered statistically significant.

## Results

### Patient Characteristics and Univariate Logistic Regression Analysis

**FIGURE 1:**
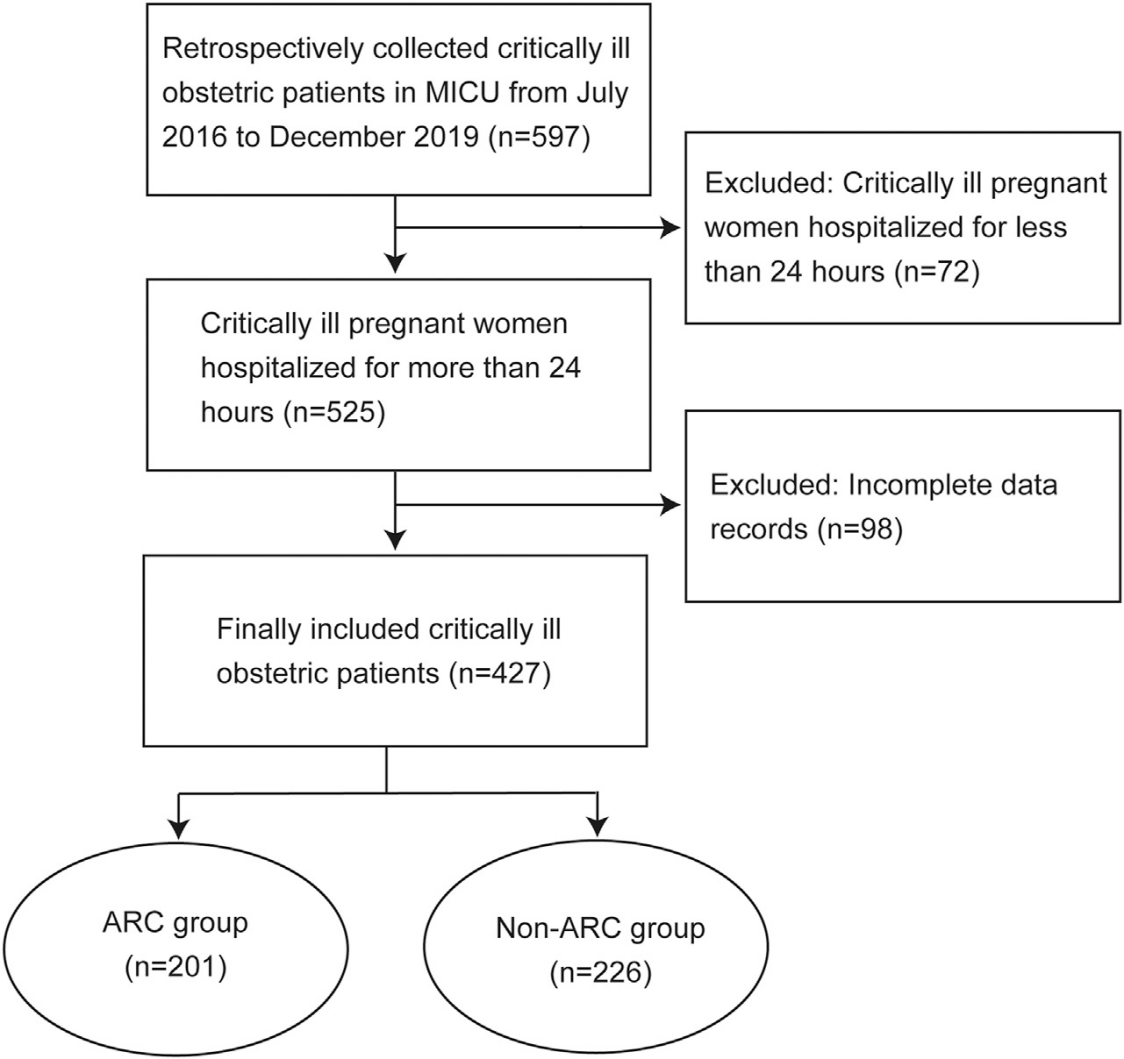
Flowcharts of patients who were included and excluded from the study.

A total of 427 critically ill obstetric patients were enrolled and divided into the ARC group (201 cases) and non-ARC group (226 cases) (Figure 1). The incidence of ARC was 47.1% (201/427). The results of univariate analysis showed that ARC group patients were taller, heavier, had greater gestational age, had higher albumin and PLT levels, had lower scores of APACHE Ⅱ, had fewer underlying conditions such as caesarean section, HELLP syndrome, hypertension and severe preeclampsia, had less use of vasoactive drug, but had more hypertriglyceridemia, infection and acute fatty liver, had statistically differences when compared with the non-ARC group (*p* < 0.05) (Table 1).

**TABLE 1:** General clinical data of patients and results of ARC risk factors screened by univariate logistic regression analysis.

Variable	Non ARC group (*n* = 226)	ARC group (*n* = 201)	*p*-value	OR	95% confidence interval
Age (y), median [IQR]	30.0 (27.0, 34.0)	29.0 (26.0, 33.0)	0.143	0.973	0.939–1.009
Height (cm), median [IQR]	160.0 (157.0, 163.0)	160.0 (158.0, 164.0)	0.007	1.058	1.015–1.101
Weight (kg), median [IQR]	67.0 (60.9, 74.0)	68.5 (62.5, 75.6)	0.003	1.031	1.011–1.052
Cr (μmol/L), median [IQR]	64.1 (56.4, 79.0)	42.0 (36.0, 47.0)	0.000	0.732	0.685–0.781
CrCL (ml/min/1.73 m^2^), median [IQR]	110.3 (87.4, 121.7)	157.8 (136.8, 204.1)	0.000	7.538	2.541–22.367
Gestational age (week), median [IQR]	33.0 (29.9, 35.4)	33.9 (30.6, 37.9)	0.002	1.074	1.027–1.122
APACHE II (score), median [IQR]	15.0 (8.2, 19.0)	10.0 (7.6, 13.8)	0.012	0.680	0.616–0.921
Hemoglobin (g/L), median [IQR]	120.0 (103.0, 130.0)	115.0 (103.0, 124.0)	0.386	0.996	0.986–1.005
PLT (×10^9^/L), median [IQR]	178.0 (122.0, 225.3)	184.0 (142.5, 238.5)	0.015	1.003	1.001–1.006
ALB (g/L), median [IQR]	28.9 (25.1, 32.3)	32.3 (28.7, 36.2)	0.000	1.081	1.047–1.117
ALT (U/L), median [IQR]	25.0 (19, 39.3)	21.0 (15.5, 33.5)	0.608	1.000	0.998–1.001
TBIL (μmol/L), median [IQR]	5.0 (1.7, 9.6)	6.2 (3.5, 11.1)	0.472	1.004	0.993–1.016
Caesarean section, n (%)	192 (85.0)	108 (53.7)	0.000	0.206	0.130–0.325
Type 2 diabetes, n (%)	54 (23.9)	43 (21.4)	0.538	0.867	0.550–1.366
Shock, n (%)	7 (3.1)	9 (4.5)	0.456	1.467	0.536–4.013
Infection, n (%)	37 (16.4)	79 (39.3)	0.000	3.308	2.105–5.198
Mechanical ventilation, n (%)	19 (8.4)	23 (11.4)	0.295	1.408	0.742–2.669
Vasoactive drug, n (%)	131 (58.0)	41 (20.4)	0.000	0.189	0.123–0.292
Diabetes ketoacidosis, n (%)	13 (5.8)	5 (2.5)	0.103	0.418	0.146–1.194
Acute pancreatitis, n (%)	10 (4.4)	17 (8.5)	0.093	1.996	0.892–4.466
HELLP syndrome, n (%)	38 (16.8)	11 (5.5)	0.000	0.286	0.142–0.577
Hypertension, n (%)	161 (71.2)	63 (31.3)	0.000	0.184	0.122–0.279
Eclampsia, n (%)	10 (4.4)	2 (1.0)	0.050	0.217	0.047–1.003
Severe preeclampsia, n (%)	159 (70.4)	49 (24.4)	0.000	0.136	0.088–0.209
Cerebral hemorrhage, n (%)	3 (1.3)	6 (3.0)	0.246	2.287	0.564–9.267
Epilepsy, n (%)	2 (0.9)	5 (2.5)	0.213	2.857	0.548–14.892
Postpartum hemorrhage, n (%)	13 (5.8)	11 (5.5)	0.171	0.544	0.228–1.300
Hypertriglyceridemia, n (%)	6 (5.3)	31 (12.4)	0.000	6.686	2.727–16.393
Uterine fibroids, n (%)	10 (4.4)	6 (3.0)	0.837	1.128	0.358–3.556
Acute heart failure, n (%)	16 (7.1)	5 (2.5)	0.548	0.847	0.492–1.457
Coronary heart disease, n (%)	3 (1.3)	7 (3.5)	0.900	0.949	0.415–2.168
Cardiac insufficiency, n (%)	12 (5.3)	3 (1.5)	0.284	0.551	0.185–1.640
Arrhythmia, n (%)	1 (0.4)	4 (2.0)	0.176	4.569	0.506–41.216
Cholestasis syndrome, n (%)	11 (4.9)	8 (4.0)	0.979	1.012	0.403–2.544
Acute fatty liver, n (%)	6 (2.7)	14 (7.0)	0.043	2.745	1.034–7.285
Asthma, n (%)	2 (0.9)	2 (1.0)	0.906	1.126	0.157–8.065
Acute respiratory failure, n (%)	7 (3.1)	7 (3.5)	0.748	0.838	0.286–2.459
Acute respiratory distress syndrome, n (%)	6 (2.7)	5 (2.5)	0.474	0.635	0.183–2.203
DIC, n (%)	6 (2.7)	5 (2.5)	0.474	0.635	0.183–2.203
Hypothyroidism, n (%)	12 (5.3)	15 (7.5)	0.364	1.438	0.657–3.150
Hyperthyroidism, n (%)	2 (0.9)	1 (0.5)	0.507	2.261	0.204–25.128

Type 2 Diabetes:fasting glucose ≥6.1 mmol/L; Hypertension:BP ≥ 140/90 mmHg; Anemia : Hb < 120 g/L; Diabetes ketoacidosis:metabolic acidosis, ketosis, and hyperglycemia; Acute pancreatitis :two out of the following three criteria: abdominal pain, lipase >3 ULN, or radiographic findings of pancreatitis on CT scan; HELLP syndrome:(1) hemolysis; (2) low platelet count; and (3) elevated liver enzymes; Pre-eclampsia:BP ≥ 140/90 mmHg after 20 weeks of gestation, proteinuria; severe preeclampsia: BP > 160/110 mmHg, new onset cerebral or visual disturbance, elevated liver enzymes, epigastric pain, pulmonary edema, low platelet count, progressive renal insufficiency; Hypertriglyceridemia: TG > 150 mg/dl; Uterine fibroids: ultrasound or MRI that suggests the presence of one or more fibroids; Acute heart failure: BNP > 100 ng/L or NT-pro-BNP > 3 00 ng/L, and LV ejection fraction ≥45%; Cholestasis syndrome: pruritus, TSBAs ≥ 10 μmol/L or ALT > 40 U/L; Acute fatty liver of pregnancy: Swansea criteria; Thrombocytopenia: PLT < 100*10^9^/L; Acute respiratory distress syndrome: defined by the Berlin definition; Acute Kidney Injury: defined using the KDIGO guidelines. ALB: Albumin; APACHE II: Acute Physiology and Chronic Health Evaluation II score; ARC, augmented renal clearance; CrCl, creatinine clearance rate; DIC, disseminated intravascular coagulation; OR:odds ratio; PLT: Platelet; TBIL: Total bilirubin.

### Multivariate Analysis and Logistic Regression Model

The risk factors for ARC were estimated by the stepwise multivariate logistic regression analysis (backward procedure, based on *p*-value of predictor removed) and the identification of the cutoff values with ROC curve analyses. Variables with *p* < 0.1 in the univariate results were estimated in the multivariate analysis (Hammitt et al., 2019). The significant independent factors for the occurrence of ARC were heavier, greater gestational age, higher albumin level, fewer caesarean section, severe preeclampsia and vasoactive drug; more infection, hypertriglyceridemia and acute pancreatitis (*p* < 0.05) (Table 2). After the introduction and elimination of the above independent risk factors independent variables, the logistic regression equation was finally established:

Logit(p)=0.056×weight+0.061×albumin+0.074×gestational age−1.000×caesarean section+0.911×infection−0.789×Vasoactive drug−1.399×Acute pancreatitis−2.950×Severe preeclampsia+2.108×hyperlipidaemia−10.804. (6)

**TABLE 2:** Results of ARC risk factors screened by multivariate logistic regression analysis.

Variable	*p*-value	OR	95% confidence interval
Height (cm)	0.404	1.024	0.969–1.082
Weight (kg)	0.000	1.057	1.025–1.091
Gestational age (weeks)	0.013	1.076	1.016–1.140
APACHE II (score)	0.450	0.635	0.343–1.178
PLT (×10^9^/L)	0.727	1.001	0.997–1.004
ALB (g/L)	0.002	1.062	1.022–1.104
Caesarean section	0.002	0.368	0.198–0.684
Infection	0.002	2.488	1.380–4.487
Vasoactive drug	0.010	0.454	0.249–0.828
Acute pancreatitis	0.033	0.247	0.068–0.894
HELLP syndrome	0.634	1.238	0.514–2.980
Hypertension	0.082	4.679	0.820–26.698
Eclampsia	0.118	0.256	0.046–1.413
Severe preeclampsia	0.001	0.052	0.009–0.296
Hypertriglyceridemia	0.003	8.228	2.048–33.062
Acute fatty liver	0.784	1.200	0.325–4.429

ALB, Albumin; APACHEII, Acute Physiology and Chronic Health Evaluation II score; OR, Odds ratio; PLT, Platelet.

### The Models’ Ability to Identify ARC Patients

**FIGURE 2:**
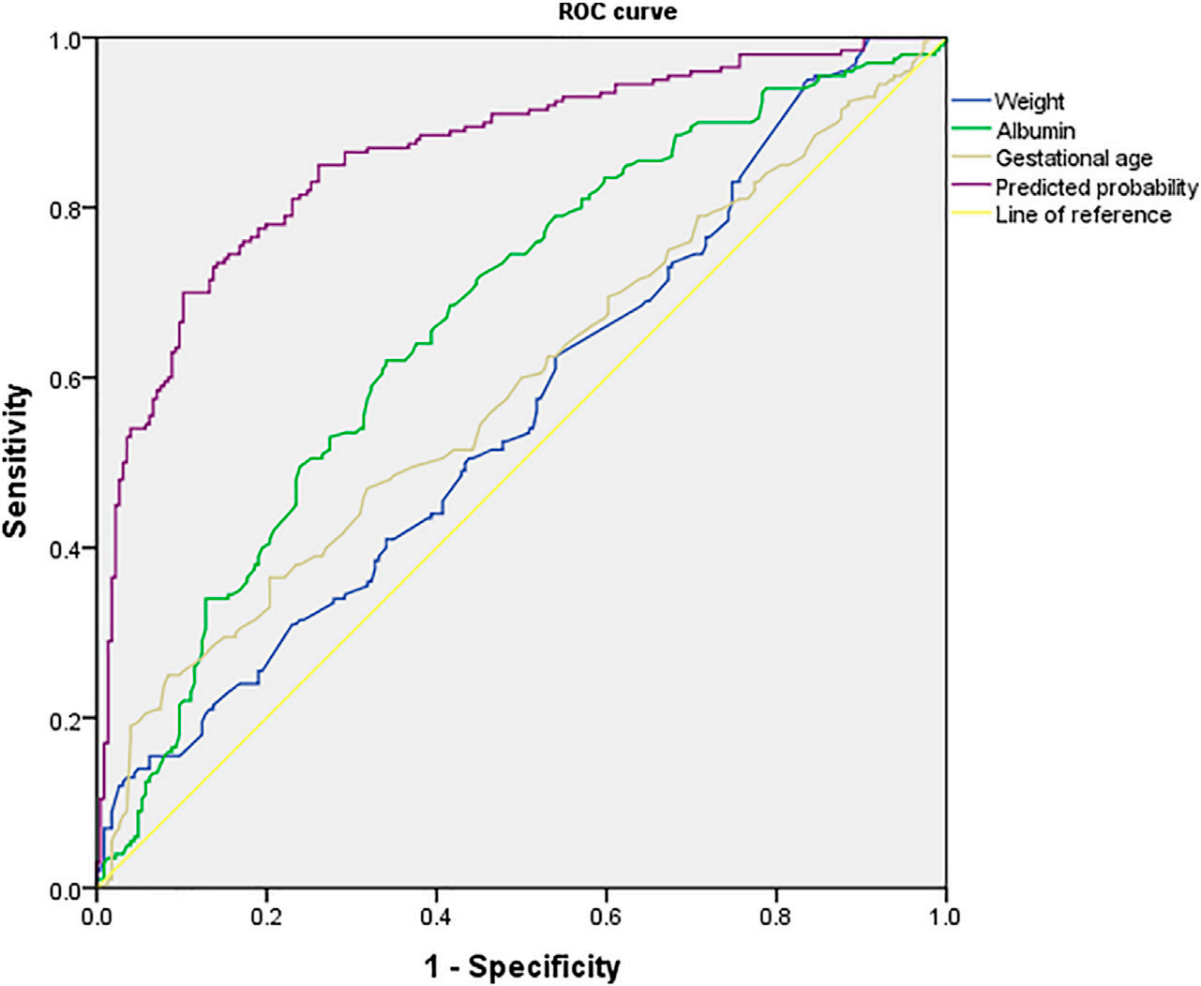
ROC curve of predicted probability, body weight, albumin and gestational age, which were independent risk factors and combined predictor for ARC. The predicted probability of combined weight (AUC = 0.563), albumin (AUC = 0.670) and gestational age (AUC = 0.587) had better sensitivity (70.0%) and specificity (89.8%), Youden index 0.598, as well as the maximal AUC (AUC = 0.863).

For each independent risk factor of continuous variables, we calculated the specificity and sensitivity of the resulting logistic regression model by constructing ROC curves and calculating the AUC to estimate the models’ ability to identify ARC patients. The ROC curve of body weight, albumin, gestational age, and predicted probability were drawn. The area under the ROC curve, cut-off point, Youden index, sensitivity, specificity of body weight, albumin, gestational age, and predicted probability were shown in Table 3 and Figure 2. The area under the ROC curve of the predicted probability was 0.863, higher than the other four factors, the Youden index was 0.598, and the sensitivity and specificity were 70.0 and 89.8%.

**TABLE 3:** Area under the curve and cut-off values of receiver operating characteristic curve for prediction of ARC in critically ill obstetric patients.

Risk factors	AUC (95%confidence interval)	*p*-value	Cut-off point	Youden index	Sensitivity (%)	Specificity (%)
Weight (kg)	0.563 (0.508–0.617)	0.026	59.5	0.114	95.0	16.4
Gestational age (weeks)	0.587 (0.533–0.641)	0.002	37.785	0.166	25	91.6
ALB (g/L)	0.670 (0.619–0.722)	0.000	30.45	0.279	62	65.9
Predicted probability	0.863 (0.827–0.898)	0.000	0.614	0.598	70.0	89.8

ALB, Albumin; AUC, Area under the curve.

### Validation of the Model and VCM Treatment in Patients

**FIGURE 3:**
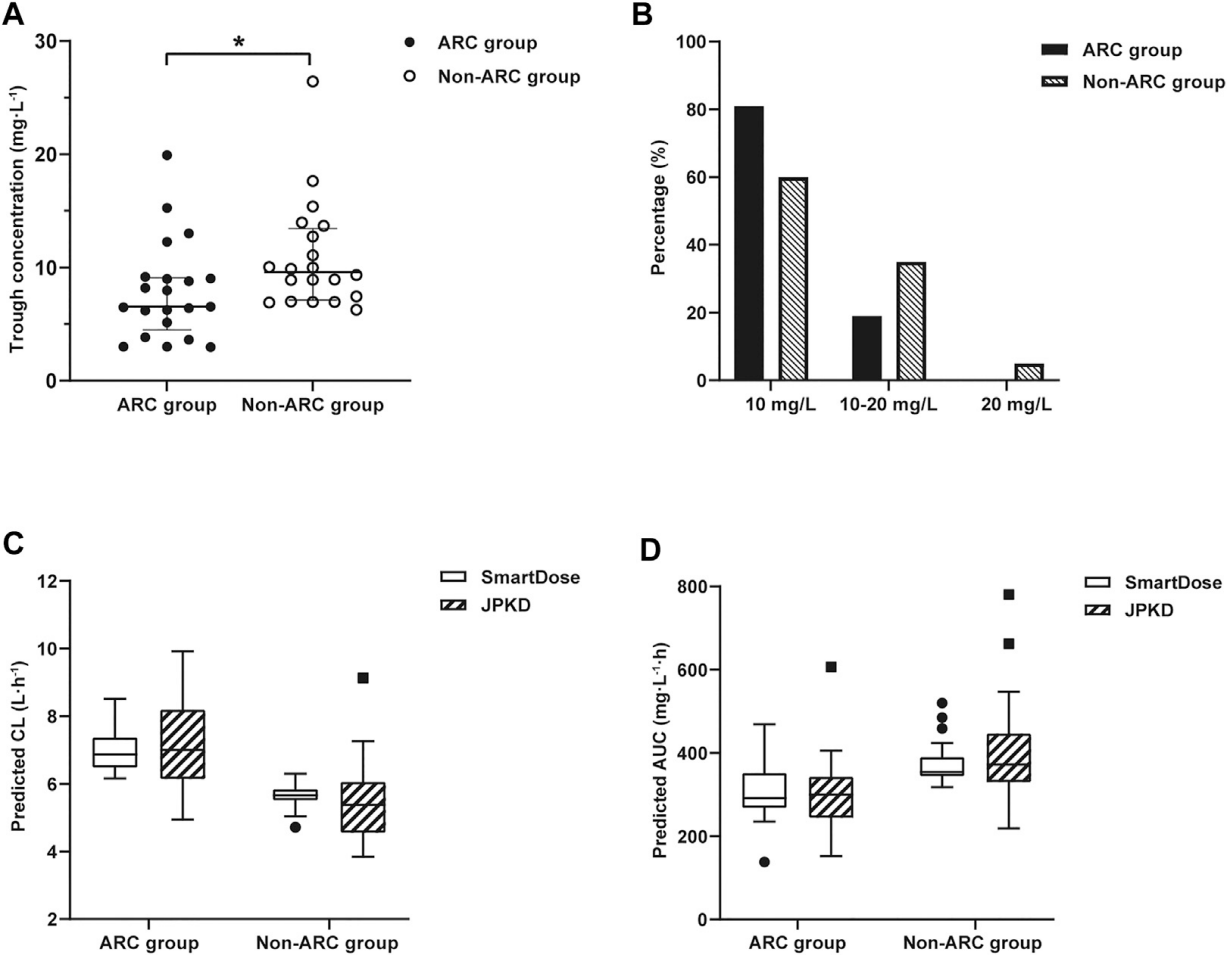
**(A)** Distribution of vancomycin initial trough concentration in ARC group and non-ARC group. Compared with the non-ARC group, trough concentration was significantly lower in ARC group (7.9 ± 3.2 mg/L vs 9.5 ± 3.3 mg/L; *p = 0.033). **(B)** The proportion of trough concentration in the target range of 10–20 mg/L or out of the range. The proportion of trough concentration<10 mg/L was 81.0% in ARC group. **(C)** Distribution of vancomycin CL in ARC group and non-ARC group. The CL of VCM calculated by *SmartDose* and *JPKD* in the ARC group were higher than that of the non-ARC group (*p* = 0.000; *p* = 0.008). **(D)** Distribution of vancomycin AUC_24 h_ in ARC group and non-ARC group. The AUC_24 h_ calculated by *SmartDose* and *JPKD* in ARC group was lower than that of non-ARC group (*p* = 0.003; *p* = 0.013).

We collected clinical data of 52 critically ill obstetric patients who received VCM therapy. Eleven patients were subsequently excluded: Six patients received VCM for less than 5 days, and five others had no steady-state trough concentrations. Finally, 41 patients were included. According to the eGFR (CKD-EPI equation), the 41 patients were divided into the ARC group (*n* = 21, 51.2%) and non-ARC group (*n* = 20, 48.8%). The predictive accuracy of this logistic regression model for ARC prediction in the ARC group and non-ARC group were 90.5 and 85.0% (Table 4). The dosage, period of VCM administration, and trough concentration were presented in Table 4. The initial trough concentration was significantly lower in the ARC group, when compared with the non-ARC group (7.9 ± 3.2 mg/L vs 9.5 ± 3.3 mg/L; *p* = 0.033) (Figure 3).

**TABLE 4:** Dosage and trough concentration of vancomycin in critically ill obstetric patients.

Groups	Prediction correct rate of model	Dose (mg/kg/d)	Period of vancomycin (d)	Trough concentration (mg/L)
ARC group (*n* = 21)	90.5%	32.7 ± 9.1	9.5 (6.5.13.0)	7.9 ± 3.2
Non-ARC group (*n* = 20)	85.0%	33.2 ± 7.3	10.0 (7.0.13.5)	9.5 ± 3.3
T/Z value	–	−0.182	−0.287	−2.213
*p*-value	0.663	0.857	0.592	0.033

ARC, augmented renal clearance.

### Predictive Performance of Vancomycin PPK Software for ARC Patients

**FIGURE 4:**
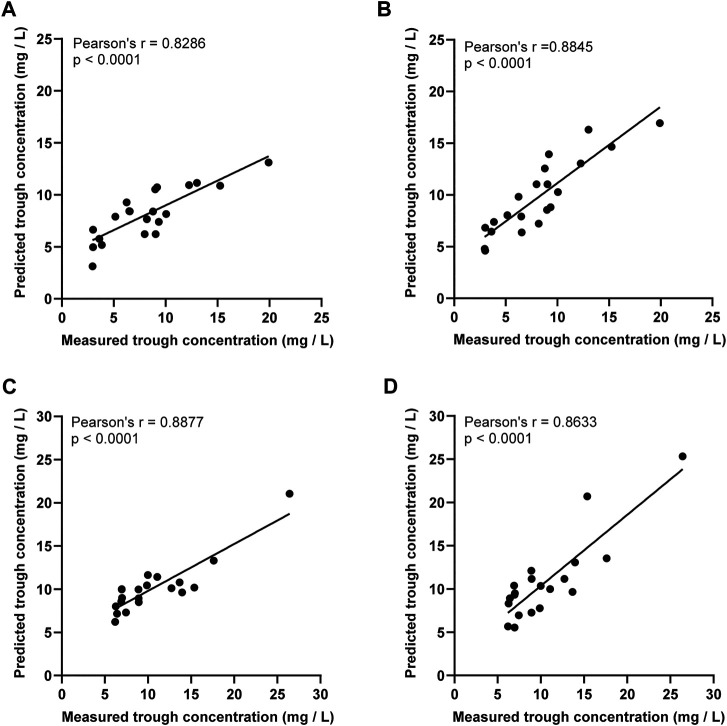
The linear regression analysis was used to compare the predicted trough concentration of vancomycin PPK software SmartDose and JPKD in ARC group and non-ARC group. **(A)** Prediction of SmartDose for trough concentration in ARC group (r = 0.8286, *p* < 0.001). **(B)** Prediction of JPKD for trough concentration in ARC group (r = 0.8845, *p* < 0.001). **(C)** Prediction of SmartDose for trough concentration in non-ARC group (r = 0.8877, *p* < 0.001). **(D)** Prediction of JPKD for trough concentration in non-ARC group (r = 0.8633, *p* < 0.001).

The therapeutic range for VCM trough concentration is 10–20 mg/L and AUC_24 h_ at 400–600 mg.h/L (MIC = 1). The distribution of vancomycin initial trough concentration and AUC_24 h_ were shown in Figure 3. There were 17 cases (81.0%) had low initial trough concentration (less than 10 mg/L) in the ARC group (Figure 3), 18 cases (85.7%) had low AUC_24 h_ (less than 400 mg.h/L) in the ARC groups. The APE of *SmartDose* and *JPKD* for prediction of initial trough concentrations had no statistical difference in the two groups (*p* = 0.443; *p* = 0.745). Comparing the predicted trough concentration of *Smartdose* and *JPKD* software with the measured trough concentration, correlation coeffcients (*r*) were 0.8286 and 0.8845 in the ARC group, and 0.8877 and 0.8633 in the non-ARC group (Figure 4). The pharmacokinetic parameters calculated by *SmartDose* and *JPKD* also had no statistical difference (Table 5). The Cl of VCM calculated by *SmartDose* and *JPKD* in the ARC group were higher than that of the non-ARC group (*p* = 0.000; *p* = 0.008), and AUC_24 h_ calculated by *SmartDose* and *JPKD* was lower than the non-ARC group (*p* = 0.003; *p* = 0.013) (Figure 3).

**TABLE 5:** Pharmacokinetic parameters of vancomycin in critically ill obstetric patients.

Groups		Predicted trough concentration (mg/L)	APE (%)	CL (L/h)	Vd (L)	AUC_24 h_ (mg·h/L)
ARC group	Smartdose	8.0 ± 2.5	28.6 (11.0, 41.6)	7.0 ± 0.6^a^	35.4 ± 1.4	309.0 ± 69.0^b^
	JPKD	9.6 ± 3.7	38.1 (10.2, 56.6)	7.2 ± 1.4^c^	40.6 ± 3.9	308.6 ± 83.1^d^
T/Z value		−1.608	−0.767	−0.967	−1.991	0.037
*p* Value		0.116	0.443	0.345	0.095	0.971
Non-ARC group	Smartdose	9.9 ± 1.7	20.6 (7.3, 28.2)	5.7 ± 0.4	37.7 ± 2.3	375.1 ± 49.2
	JPKD	10.3 ± 3.9	20.8 (6.4, 33.8)	5.6 ± 1.3	39.2 ± 3.8	387.9 ± 104.6
T/Z value		−0.689	−0.325	0.349	−1.687	−1.333
*p* Value		0.495	0.745	0.731	0.112	0.198

APE, Absolute prediction error; ARC, augmented renal clearance; CL: Clearance; Vd, Volume of distribution; AUC_24 h_, Area under the curve.

Compared with non-ARC group,

a p = 0.000

b p = 0.003

c p = 0.008

d p = 0.013

## Discussion

Observational studies show that ARC is present in 20–65% of critically ill patients, and that it seems to be more common in patients with burns or traumatic brain injury (Bilbao-Meseguer et al., 2018). Our study population is critically ill obstetric patients, having similar young age, heavier weight, and fewer underlying diseases. In our study, the frequency of occurrence of ARC in critically ill obstetric patients was 47.1%, suggesting that ARC is more common in obstetric patients. Studies have reported that polytrauma, younger age, lower illness severity and other factors like brain injury, male sex, less vasopressor use, and increased cardiac index are independent determinants of ARC in critically ill patients (Bilbao-Meseguer et al., 2018). But in our study, the risk factors are different from other critically ill patients. Therefore, there is a need to establish a logistic regression model to predict ARC in obstetric patients.

According to the results of univariate and multivariate logistic regression analysis, we established a nine-variables regression model. The independent risk factors positively associated with ARC were weight, gestational age, albumin level, infection, hypertriglyceridemia and acute pancreatitis. The CKD-EPI equation was used to calculate the eGFR in our study, it is indexed relative to an average body surface area of 1.73 m^2^ and expressed as mL/min/per 1.73 m^2^ (Levey et al., 2015). GFR varies by body size, the formula of body surface area includes height and weight, so the weight has a direct effect on eGFR. Zhou Q et al. analyzed that body mass index (BMI) and serum creatinine level were independent predictors of ARC (Zhou et al., 2020). The formula of BMI includes weight and height, so the weight was related with ARC. Kawano et al. (Kawano et al., 2016) also analyzed the associated factors of ARC in Japanese ICU patients; the results showed that body weight is heavier in ARC patients, when compared with patients without ARC (*p* < 0.05). So far, no direct association between ARC and albumin or gestational age has been reported. Increased gestational age and higher albumin level means weight gained and better nutrition, which maybe means the patients are more likely to develop ARC. The incidence of ARC is higher in obstetric patients with hypertriglyceridemia and acute pancreatitis. Hypertriglyceridemia may be the cause of important disease in pregnant patients. Patients with triglyceride levels exceeding 1,000 mg/dl are at increased risk of developing severe pancreatitis (Cruciat et al., 2020). Some studies reported that 54.1% (33/61) of the SAP patients had a high CrCl (more than 130 ml/min/1.73 m^2^), and the serum trough concentration of vancomycin was significantly reduced in SAP patients with ARC (He et al., 2016; He et al., 2017). In our study, ARC patients had more infection but less use of vasoactive drug, which may be related to systemic inflammatory response and capillary leak, resulting in a greater Vd, which is similar to literature reports (He et al., 2017; Bilbao-Meseguer et al., 2018).

On the other hand, the independent risk factors negatively associated with ARC were caesarean section, severe pre-eclampsia and vasoactive drug in our study. ARC patients had lower APACHE II scores and fewer basic diseases in univariate logistic regression analysis, such as after operation, hypertension, which is similar to research reports (Hobbs et al., 2015; Bilbao-Meseguer et al., 2018; Mulder et al., 2019). So far, no direct association between ARC and eclampsia, or HELLP syndrome has been reported. HELLP syndrome is one of the serious complications of severe preeclampsia. Pregnant patients with preeclampsia have aggravated renal vasospasm, which causes renal ischemia and hypoxia and aggravates renal function injury. Patients with HELLP syndrome and eclampsia in later stage are at increased risk of acute kidney damage. The occurrence of AKI is associated with infection, severe hypertension, and renal ischemia; elevated creatinine is an independent predictor of maternal mortality in HELLP syndrome (Ye et al., 2019).

According to the risk factor analysis, many studies have established ARC detection methods, like a scoring method or model (Bilbao-Meseguer et al., 2018; Saito et al., 2020). In our study, ROC curves for single risk factor indicated that the predictive value is not significant, so we established a regression model with nine factors to identify the ARC in critically ill obstetric patients. The predicted probability of this model had an AUCROC of 0.866 (*p* < 0.001); sensitivity and specificity were 93.8 and 54.2%, which means that the model had a high predictive value. Verification of 41 other critically ill obstetric patients treated with vancomycin showed that the predictive accuracy of this model for ARC prediction was more than 80%, which is a high predictive performance.

Previous studies have shown that the blood levels of VCM are reduced in ARC patients, and it is recommended to perform TDM or administer high doses (Chu et al., 2016; Hirai et al., 2016; Tang et al., 2018a; Tang et al., 2018b). The trough concentration of vancomycin is highly correlated with the clinical efficacy, and TDM helps improve the effectiveness and safety of vancomycin therapy (Rybak et al., 2020). In our study, the trough concentration of vancomycin was significantly lower in the ARC group, similar to what has been found in other research studies (Chu et al., 2016; Hirai et al., 2016; Tang et al., 2018a; Tang et al., 2018b). ARC also affects the pharmacokinetics of VCM: our results exhibited higher CL than the non-ARC patients, and the AUC_24 h_ was lower than for non-ARC patients, but Vd had no significant difference, in agreement with the literature reports (Hirai et al., 2016). These changes in pharmacokinetic parameters partly explained the reason why the trough concentration of VCM in critically ill obstetric ARC patients was lower than that in non-ARC patients. For this special group of patients, a higher VCM dose should be considered to achieve the target concentration of VCM. The TDM guidelines of vancomycin recommend the use of PPK in the design of individualized dosage regimen (Rybak et al., 2020). We evaluated the predictive performance of vancomycin PPK software *SmartDose* and *JPKD* for predicting trough concentration and AUC_24 h_. Our results indicated that the precision of *SmartDose* and *JPKD* for prediction of initial trough concentrations had no statistical difference between the ARC and non-ARC group (*p* = 0.309; *p* = 0.775). The results of linear regression analysis indicated that *SmartDose* and *JPKD* had a good predictive performance for initial trough concentrations in ARC patients. *SmartDose* and *JPKD* can provide reference for the individualized dosage regimen of vancomycin in ARC patients.

However, there are a few limitations of this study: (1) Our study analyzed risk factors of ARC in critically ill obstetric patients, but it is possible that not all impact factors were included. (2) The entire data came from the perinatal medical database, which is representative of the Suzhou area, but biases may still exist. (3) In this study, the CLcr was determined using the CKD-EPI equation, which is not as accurate as direct measurement of creatinine in urine, which may lead to the inaccurate correlation coefficient between the creatinine clearance rate and VCM trough concentrations. (4) This is a single-center clinical study, so the sample of critically ill obstetric patients treated with VCM is too limited to precisely estimate the pharmacokinetics of VCM. In the future, multicenter clinical cohort studies can be undertaken with more impact factors included to analyze and evaluate risk factors of ARC in critically ill obstetric patients and the influence of ARC on VCM pharmacokinetics and clinical efficacy.

## Conclusion

ARC is a common state in critically ill obstetric patients. The regression model had high prediction value for predicting ARC. The pharmacokinetic parameters of VCM in the ARC patients are different from those of other groups, which resulted in enhanced VCM clearance and decreased drug trough concentration. Vancomycin PPK software *JPKD* and *SmartDose* both had good predictive performance for predicting trough concentrations of ARC and non-ARC obstetric patients.

## Data Availability

The raw data supporting the conclusion of this article will be made available by the authors, without undue reservation.
